# An increased burden of rare exonic variants in *NRXN1* microdeletion carriers is likely to enhance the penetrance for autism spectrum disorder

**DOI:** 10.1111/jcmm.16161

**Published:** 2021-01-21

**Authors:** Cinzia Cameli, Marta Viggiano, Magali J. Rochat, Alessandra Maresca, Leonardo Caporali, Claudio Fiorini, Flavia Palombo, Pamela Magini, Renée C. Duardo, Fabiola Ceroni, Maria C. Scaduto, Annio Posar, Marco Seri, Valerio Carelli, Paola Visconti, Elena Bacchelli, Elena Maestrini

**Affiliations:** ^1^ Department of Pharmacy and Biotechnology University of Bologna Bologna Italy; ^2^ UOSI Disturbi dello Spettro Autistico, Ospedale Bellaria di Bologna IRCCS Istituto delle Scienze Neurologiche di Bologna Bologna 40139 Italy; ^3^ IRCCS Istituto delle Scienze Neurologiche di Bologna UOC Clinica Neurologica Bologna Italia; ^4^ Department of Biomedical and Neuromotor Sciences (DIBINEM) University of Bologna Bologna Italy; ^5^ Unit of Medical Genetics Department of Medical and Surgical Sciences Policlinico St. Orsola‐Malpighi Hospital University of Bologna Bologna Italy; ^6^ Faculty of Health and Life Sciences Oxford Brookes University Oxford UK

**Keywords:** ASD, mtDNA, *NRXN1*, penetrance, rare variants

## Abstract

Autism spectrum disorder (ASD) is characterized by a complex polygenic background, but with the unique feature of a subset of cases (~15%‐30%) presenting a rare large‐effect variant. However, clinical interpretation in these cases is often complicated by incomplete penetrance, variable expressivity and different neurodevelopmental trajectories. *NRXN1* intragenic deletions represent the prototype of such ASD‐associated susceptibility variants. From chromosomal microarrays analysis of 104 ASD individuals, we identified an inherited *NRXN1* deletion in a trio family. We carried out whole‐exome sequencing and deep sequencing of mitochondrial DNA (mtDNA) in this family, to evaluate the burden of rare variants which may contribute to the phenotypic outcome in *NRXN1* deletion carriers. We identified an increased burden of exonic rare variants in the ASD child compared to the unaffected *NRXN1* deletion‐transmitting mother, which remains significant if we restrict the analysis to potentially deleterious rare variants only (*P* = 6.07 × 10^−5^). We also detected significant interaction enrichment among genes with damaging variants in the proband, suggesting that additional rare variants in interacting genes collectively contribute to cross the liability threshold for ASD. Finally, the proband's mtDNA presented five low‐level heteroplasmic mtDNA variants that were absent in the mother, and two maternally inherited variants with increased heteroplasmic load. This study underlines the importance of a comprehensive assessment of the genomic background in carriers of large‐effect variants, as penetrance modulation by additional interacting rare variants to might represent a widespread mechanism in neurodevelopmental disorders.

## 1 INTRODUCTION

Autism Spectrum Disorder (ASD) is a heterogeneous neurodevelopmental disorder with a high prevalence (>1%) and a remarkable social burden, with no effective pharmacological treatments.^1^ Despite the high heritability, the vast majority of genetic risk factors still remains unknown and only about 15%‐30% of ASD individuals have an identifiable genetic cause.^2^

Several years of investigation has led to considerable progress in the identification of a large number of risk genes and the delineation of a heterogeneous and complex genetic architecture. It is now evident that ASD, like other neuropsychiatric disorders, has a polygenic basis, but with the peculiarity of a subset of cases where a large‐effect variant is present. Advance in microarrays and whole‐exome sequencing (WES) enabled the discovery of many rare variants of large effect, both structural and single‐nucleotide variants, which pinpointed more than 100 high‐confidence specific genes and genetic loci.^3^, ^4^, ^5^ Even if the identification of rare genic likely pathogenic mutations can be extremely informative, their translation into clinical settings is not straightforward, as many of them are characterized by incomplete penetrance and variable expressivity. It is possible that these rare large‐effect variants may act affecting neurodevelopmental processes common to different disorders and the final clinical manifestation rely on the presence of additional risk variants that allow crossing the threshold for clear neuropsychiatric disorders, ultimately defining the specific phenotypic trajectory in the carrier. There are several examples where the penetrance of a ‘pathogenic’ copy number variants (CNV) is increased by the presence of additional risk CNVs.^6^

Rare intragenic deletions affecting Neurexin‐1 (*NRXN1*) represent the prototype example of susceptibility variants for neurodevelopmental disorders with such complexity.^7^, ^8^ *NRXN1* belongs to the neurexin family encoding for an evolutionarily conserved presynaptic cell adhesion molecules involved in formation and maintenance of synaptic connections and vesicular neurotransmitter release. *NRXN1* is transcribed in neurons from two independent promoters which generate a longer alpha (α) and a shorter beta (β) isoform, composed of distinct extracellular domains but with an identical intracellular sequence. Moreover, through extensive alternative splicing, thousands of isoforms are produced and differentially expressed throughout the brain, with a likely role as surface recognition molecules that specify synapses.^9^ The *NRXN1* locus is extremely prone to non‐recurrent deletions with different size and breakpoint location, resulting from chromosomal rearrangements due to genomic instability. Rare intragenic deletions spanning *NRXN1* have been described in individuals with ASD, Attention‐Deficit/Hyperactivity Disorder (ADHD), intellectual disability (ID), epilepsy, schizophrenia and bipolar disorder, but also in unaffected parents, siblings and healthy controls, suggesting reduced penetrance and the contribution of other interacting genetic and/or environmental factors that influence clinical features and severity.^7^, ^8^ The enhanced frequency of additional pathogenic CNVs in cohorts of patients carrying *NRXN1* deletions has added support to the role of secondary and independently segregating genetic risk factors in the definition of the final phenotype in children with inherited deletions. Previous studies suggested that deletion extent and exon content may also play a role in this clinical heterogeneity; however, there is no consensus on the different penetrance of 5′ *NRXN1* deletions (exons 1‐6, NM_001135659.2) versus 3′ *NRXN1* deletions (exons 7‐24, NM_001135659.2). Specifically, one study proposed a lower penetrance of 3′ *NRXN1* deletions as they are more frequently co‐occurring with another rare and often pathogenic CNV,^7^ while a more recent study reported a higher penetrance of 3′ deletions, given the much higher frequencies in cases versus controls and the higher de novo occurrence.^8^

In order to identify pathogenic CNVs in ASD probands, we performed array‐based comparative genomic hybridization (aCGH) on a cohort of 104 ASD individuals from 89 Italian families. A 3′ *NRXN1* deletion was identified in a trio family in which a girl with ASD inherited the deletion from the unaffected mother. To further explore the impact of genetic background on the penetrance of *NRXN1* deletions, a detailed clinical evaluation of the girl and her parents was combined with genetic analysis including (a) WES, in order to characterize the background of rare variants and (b) deep sequencing of the entire mitochondrial genome, with the aim of detecting rare pathogenic mutations and evaluating the burden of low‐heteroplasmy variants. Mitochondrial DNA (mtDNA) defects could, indeed, represent an overlooked contributing factor to ASD susceptibility, by reducing mitochondrial function sufficiently to fall below the brain's bioenergetics threshold.^10^ Moreover, a recent study has provided evidence for a coordinated down‐regulation of synaptic and mitochondrial function genes in post‐mortem brain of ASD subjects,^11^ suggesting that a mitochondrial dysfunction might enhance the clinical outcome of *NRXN1* deletions.

## 2 EXPERIMENTAL SECTION

### 2.1 Participants

A total of 89 Italian families with one or more children with an ASD diagnosis were recruited at the UOSI Disturbi dello Spettro Autistico, IRCCS Istituto delle Scienze Neurologiche (Bologna, Italy).

Assessment and a deep phenotypic characterization of probands were done using a set of standardized clinical tests to evaluate the presence and severity of ASD (ADOS, CARS and M‐CHAT), to assess both developmental/cognitive levels (PEP‐3, Leiter‐R, Griffith Scales, or Wechsler Scales) and adaptive behaviour (Vineland Adaptive Behavior Scale, VABS) as well as discrete and clinical signs like mimicry, hyperactivity, sensory abnormalities and symptoms onset. Moreover, probands underwent EEG and MRI. Subclinical features in relatives were assessed using the Social and Communication Disorders Checklist and The Broad Autism Phenotype Questionnaire.

The ASD sample includes 78 males and 26 females, from 18 multiplex and 71 simplex families. DNA samples from both proband's parents were available for 87 families, and from a single parent for the remaining ones. All DNA samples were extracted from whole blood.

All participants provided a written informed consent to participate to this study. This study was approved by the local Ethical Committee (Comitato Etico di Area Vasta Emilia Centro (CE‐AVEC); code CE 14060). All research was performed in accordance with the relevant guidelines and regulations.

### 2.2 Copy number variant analysis

Array‐based comparative genomic hybridization (aCGH) was performed on 104 ASD individuals (89 probands, 13 siblings, 1 cousin and one uncle) and 5 siblings with another neurodevepmental disorders, using the SurePrint G3 Unrestricted CGH ISCA v2, 8x60K (Agilent Technologies) or the SurePrint G3 Human CGH Microarray 8x60K (Agilent Technologies), following manufacturer's instructions. Scan images were analysed through the Agilent CytoGenomics 5.0.1.6 software and aberrations were called by the ADM1 algorithm with a threshold of 6.0 and at least three consecutive oligonucleotides with similar log2 ratios.

All CNVs were compared to those collected in different public databases: Database of Genomic Variants (DGV, http://projects.tcag.ca/variation/), DECIPHER (https://decipher.sanger.ac.uk/), ClinVar (https://www.ncbi.nlm.nih.gov/clinvar/), and database of human CNVs hosted by IRCCS OASI Maria SS. (Troina, Italy). The American College of Medical Genetics (ACMG) guidelines were used for CNV interpretation and reporting.^12^ According to these criteria, the only identified pathogenic CNV is a deletion involving the *NRXN1* gene in a girl with ASD from a simplex family. Parental inheritance and validation of *NRXN1* deletion were carried out with quantitative PCR (qPCR) using SsoAdvanced™ Universal SYBR^®^ Green Supermix (BIORAD). The assay was performed in triplicate, with four sets of primers corresponding to the region of interest and another mapping to a control region on FOXP2 gene at 7q31.1. The number of copies of each amplified fragment was calculated using the ddCt method.

### 2.3 Whole‐exome analysis

DNA from ASD proband and parents was subjected to exome capture using NimbleGen SeqCap EZ MedExome enrichment kit (Roche), followed by paired‐end reads sequencing on an Illumina NextSeq550 (Illumina Inc, San Diego, CA, USA). Exomes had a read depth (DP) of 10× or more for 90% of the total exome coverage and 20x or more for 80%. Coverage statistics and comparison of coverage between samples was performed using QualiMap,^13^ showing a mean coverage depth of 120‐122X for the three samples and a mean quality mapping is 58. Data analysis was performed using CoVaCS,^14^ a pipeline exploiting a consensus call‐set approach from three different algorithms (GATK, Varscan and Freebayes) to generate a final set of high‐confidence variants. All variants were annotated with ANNOVAR, using RefSeq for gene‐based annotation (position, nomenclature, gene name, gene function). In order to remove low‐quality variants, genotypes were required to have DP ≥ 10, and Genome Quality (GQ) ≥20. A minor allele frequency (MAF) threshold of ≤0.5% in gnomAD exome (https://gnomad.broadinstitute.org/)^15^ (and <1% in gnomAD genome and the 1000 Genomes Project^16^) was chosen. We selected exonic and splicing variants, excluding synonymous variants. Likely deleterious variants were prioritized to capture Likely Gene Disrupting (LGD) and damaging missense mutations. LGD mutations include stop‐gain, stop‐loss, frameshift and splicing mutations, while missense mutations were defined damaging if they satisfy at least two of the following criteria: SIFT ≤ 0.05, Polyphen2 (HDIV) ≥ 0.95, Mutation Assessor score ≥ 2, CADD ≥ 15, placental mammal PhyloP ≥ 2.4 and vertebrate PhyloP ≥ 4.^17^ Mutation intolerant genes were defined by two metrics: residual variance to intolerance score (RVIS ≤ 20th percentile)^18^ and probability of loss‐of‐function intolerance (pLI score ≥ 0.9).^19^ To select genes previously associated with ASD, we used the SFARI gene database and its scoring system, including 4 categories: S (syndromic), 1 (high confidence), 2 (strong candidate) and 3 (suggestive evidence) (https://gene.sfari.org/, Release: 2019 Q4). Brain expressed, synaptic and postsynaptic density (PSD) genes were defined as previously described.^20^, ^21^, ^22^ Likely damaging de novo variants have been validated by Sanger sequencing method.

### 2.4 Deep sequencing of mitochondrial genome

Direct sequence analysis of the entire mtDNA molecule was performed on total DNA extracted from blood, by next generation sequencing (NGS) approach.^23^ Briefly, the mitochondrial genome was amplified in two long‐range PCR, the NGS library constructed by Nextera XT (Illumina) and paired‐end sequenced on NextSeq Instrument (Illumina), using an High Output Kit (300 cycle). Fastq files were analysed with an in‐house pipeline, integrating three different callers (MToolBox, Unified Genotyper of GATK and DetermineVariants)^24^, ^25^, ^26^ to detect low‐level heteroplasmy.

### 2.5 Mitochondrial DNA quantification

MtDNA content was assessed on total DNA extracted from blood, using a multiplex probe‐based real‐time PCR method,^27^ co‐amplifying a mitochondrial gene (MT‐ND2) and a nuclear gene (FASLG). All three individuals were compared with age‐matched control groups of healthy individuals.

## 3 RESULTS

### 3.1 Comparative genomic hybridization array CGH

Comparative genomic hybridization array CGH was performed in 104 individuals with ASD.

**Figure 1:**
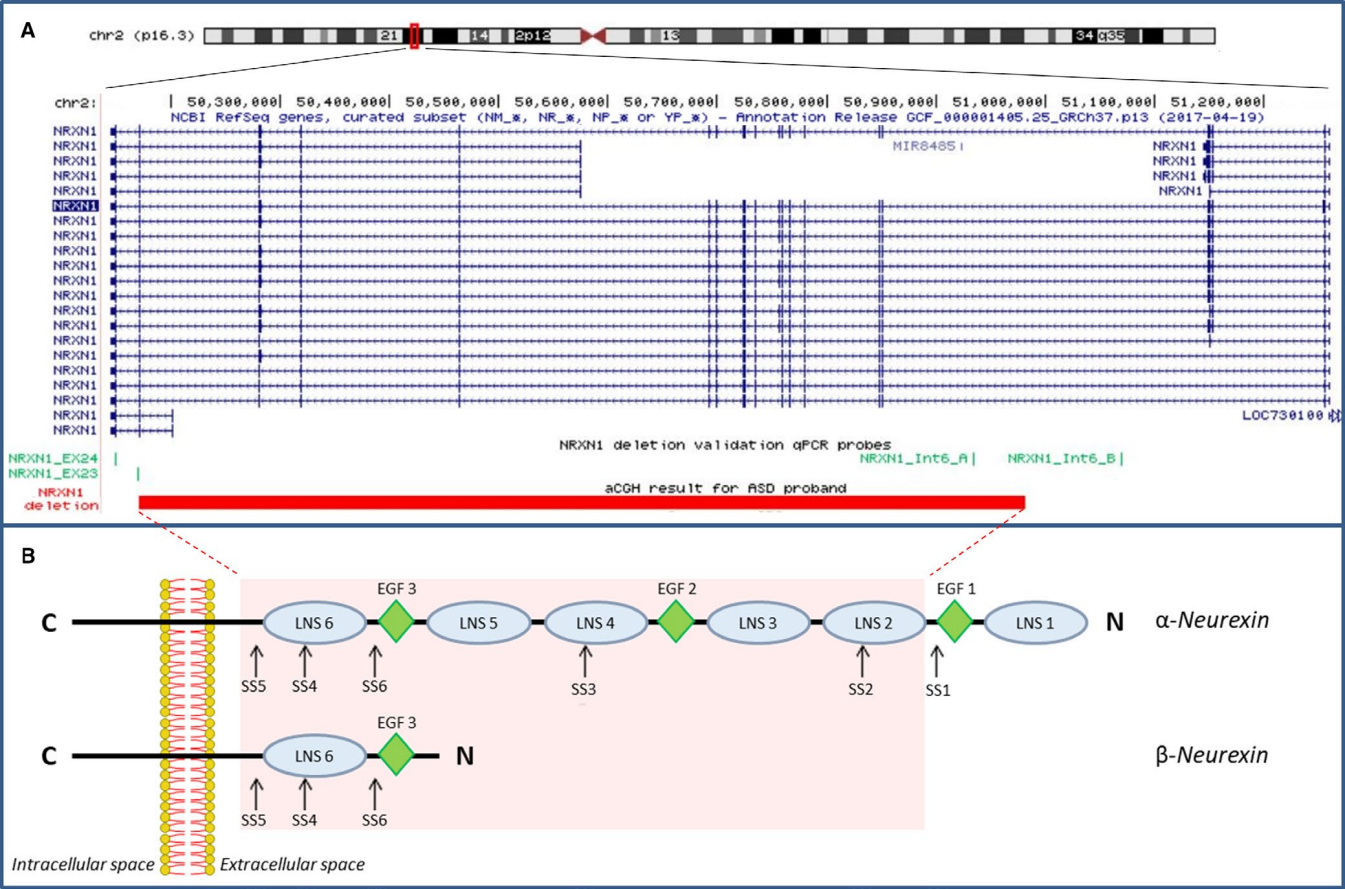
*NRXN1* deletion in the ASD proband. A, UCSC hg19 screenshot showing the *NRXN1* maternally inherited deletion detected by array CGH in the female proband and validated by qPCR in the family. qPCR probes used for validation and inheritance testing are shown in green. B, Schematic representation outlining domains structure of neurexin alpha and neurexin beta protein variants. Canonical splice sites (SS) of neurexins are indicated by arrows. Protein region affected by deletion is highlighted in red

The only pathogenic CNV identified was a deletion of ~811 kb at 2p16.3 (NC_000002.11:g.50170766_50982172del) (Figure 1A) involving exons from 7 to 23 of the *NRXN1* gene (NM_001135659.2) in a trio family. qPCR in all family members confirmed the presence of the *NRXN1* intragenic deletion in the proband and showed that the CNV is inherited from the unaffected mother (Figure S1). We identified three other rare CNVs in this family (Table S1), but none of them is considered to be clinically relevant.

### 3.2 Clinical characterization of the family with the *NRXN1* deletion

No familiarity for ASD, congenital malformations or intellectual disability was reported. The female proband was born without pre‐, peri‐ or post‐natal relevant findings. Birth weight was 3690 kg. Development of socio‐communicative and motor abilities was reported to be slightly delayed until 18 months of age with acquisition of some words. At 18‐19 months of age, parents reported a regression of the acquired socio‐communicative abilities: the girl stopped responding to simple commands and to her name. Eye‐contact was lacking, while social isolation started to be more evident. Imitation skills, communicative gestures and language stopped. She started to show hyperactivity, short attention span and motor stereotypies such as hand flapping when excited. She manifested sensory interests manipulating materials mostly to get visual, acoustic and tactile stimulation (ie passing a hair or thread upon her lips, thread waving and ripping paper in thin stripes) and a restricted interest for hair and threads. At 4.6 years old, language expression was limited to 4 single words, while language comprehension seemed to be relatively better. Hyperactivity appeared to be slightly reduced. No epileptic seizures were reported. Diagnosis of ASD was made at 35 months old through clinical observation, the Childhood Autism Rating Scale‐Second edition (CARS2‐ST)^28^ and the Autism Diagnostic Observation Schedule (ADOS‐2: module 1)^29^ (Table 1). Both diagnostic tests indicated the presence of severe clinical signs of autism. No cognitive or psychomotor development level could be assessed using standardized scales due to the lack of the child's compliance. At 4.8 years of age, the assessment of adaptive behaviour (Vineland Adaptive Behavior Scale)^30^ showed a significant delay in Communication, Daily Living Skills, Socialization and Motor Skills domains (age equivalent = 1.6 years old). Neurological examination showed: macrocrania, speech delay, aloneness, stereotypies as turning around herself, and no neuromotor signs. The proband underwent an awake and sleep EEG at the age of 2.8 years old, showing only an unspecific predominance of a slow background activity in the right temporal regions. Parents did not provide consent to perform a brain MRI.

**Table 1:** Summary of clinical data

	Mother	Father	Proband
Age at first assessment	NA	NA	23 mo
Sex	F	M	F
Microarray	*NRXN1* del/+	*NRXN1* +/+	*NRXN1* del/+
Morphology			
Growth at birth	NA	NA	Weight = 3.69 kg (50%ile); length = 56 cm (85%‐97%ile)
Head circumference (%ile)	NA	NA	Birth = 38.2 cm (>98%ile); 31 mo = 53 cm (>98%ile); 60 mo = 54.5 cm (>98°perc.)
Neurodevelopment			
Full‐scale IQ	NA	NA	NA‐NC
Speech delay	‐	‐	+
Adaptive level of functioning*	NA	NA	Vineland Adaptive Behavior Scales: CA: 4.8 y; Global AE: 1.6 y
Clinical diagnosis	NA	NA	Autism spectrum disorder
Autism scales			
CARS2‐ST	NA	NA	Total score = 46 (severe autistic symptoms) Autism cut‐ off: 30
ADOS‐2 (module 1)	NA	NA	Total score = over Autism cut‐off, Comparison score = 10 (severe autism)
Broad autism phenotype			
Broad Autism Phenotype Questionnaire (BAPQ)	Within normal range, Scores under cut‐off for Aloof , Rigid, and Pragmatic language	Within normal range, Scores under cut‐off for Aloof , Rigid, and Pragmatic language	NA
Social and Communication Disorder Checklist (SCDC)	T = 4 (<cut‐off: 9)	T = 2 (<cut‐off:9)	NA
Neurological			
EEG	NA	NA	Aspecific abnormalities: slow activity in right temporal regions
Congenital			
Other medical	‐	stuttering	‐

Abbreviations: ±, positive/negative for attribute; AE, age equivalent; CA, chronological age; NA, information not available; NC, non‐collaborative patient.

Both parents were evaluated for the presence of subclinical neurocognitive or neuropsychiatric features. Scores for the broad autism phenotype were in the normal range (Social and Communication Disorders Checklist and The Broad Autism Phenotype Questionnaire^31^, ^32^). To contextualize their phenotypic profiles beyond questionnaires, we also investigated their education status: they both reached a good education level (high school diploma and bachelor's degree, respectively) with no need for special education or services. No family history of psychiatric disorders was present, except for the paternal grandfather who was reported to take depression medication.

### 3.3 Whole‐exome sequencing

A trio‐based whole‐exome sequencing (WES) approach was undertaken for this family. We focused our analysis on rare variants (MAF ≤ 0.5%), and more specifically on those predicted to have a functional effect, including LGD variants and missense variants defined damaging, according to a combination of prediction algorithms.^17^ We compared the load of rare variants between the proband and her mother: the proband has a higher number of rare variants compared with the *NRXN1* deletion‐transmitting mother (1036 versus 573). This difference remains significant by considering the putative damaging variants only (303 in the proband vs 212 in the mother, χ^2^ = 16.08, *P* = 6.07 × 10^−5^). We then tested for transmission disequilibrium of damaging variants from the parents to the proband and we detected a preferential transmission of damaging variants from the father (203 transmitted vs 165 untransmitted variants, TDT *P* = 0.048) but not from the mother.

**Figure 2:**
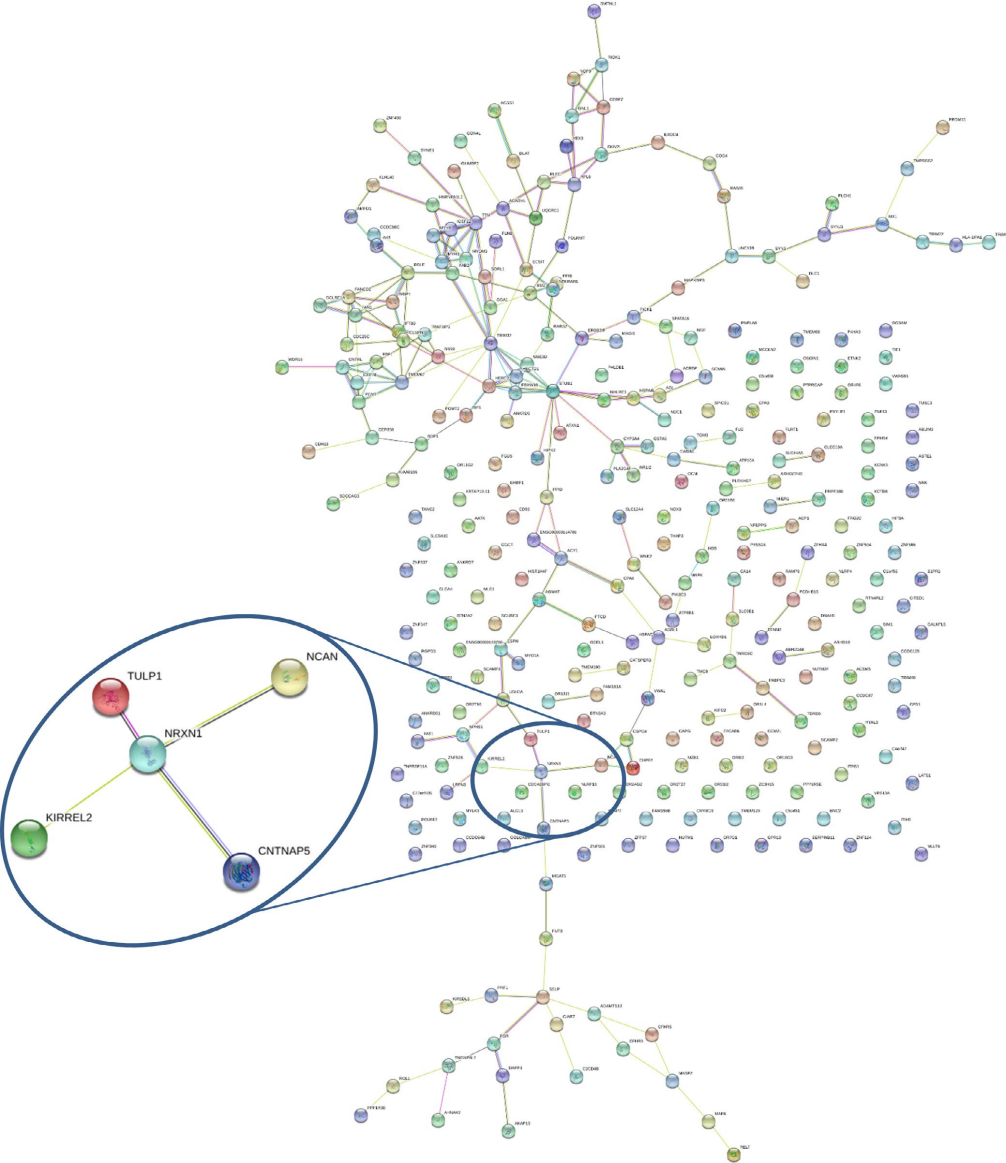
STRING network of predicted protein‐protein interactions for genes harbouring LGD or likely damaging missense variants identified by WES in the female proband. The network of predicted NRXN1 associations has been magnified. The edges represent the predicted functional associations and line colour indicates the type of interaction evidence: Red line—presence of fusion evidence; Green line—neighbourhood evidence; Blue line—cooccurrence evidence; Purple line—experimental evidence; Yellow line—text‐mining evidence; Light blue line—database evidence; Black line—co‐expression evidence. NRXN1 and CNTNAP5 show homology, co‐expression and text‐mining interaction evidence; NRXN1 and KIRREL2 show text‐mining interaction evidence; NRXN1 and NCAN show co‐expression and text‐mining interaction evidence; NRXN1 and TULP1 show co‐expression and experimental interaction evidence

Genes with at least one LGD or putative damaging missense variant or CNV were analysed using the STRING database^33^ to test the presence of an enrichment for functionally related networks of genes (Figure 2). This analysis identified a significant 1.2‐fold enrichment (223 edges vs 184 expected) in interactions among the 294 genes identified in the proband (*P* = 0.003, one‐tailed hypergeometric test), while no significant interaction enrichment was identified in *NRXN1* deletion‐transmitting unaffected mother (84 edges vs 86 expected, *P* = 0.62).

Next, we looked for specific sequence variants identified in the proband that might contribute to the clinical manifestation of the *NRXN1* deletion in the affected girl compared with the unaffected carrier mother. We first tested the possible presence of compound heterozygosity in *NRXN1*, but we found no rare functional nucleotide variants on the non‐deleted allele. Then, we focused on four categories of rare variants: (a) likely damaging de novo variants, (b) recessive‐acting variants (homozygous and compound heterozygous variants), (c) likely deleterious variants in genes previously implicated in ASD (SFARI genes) that are intolerant to functional variation (RVIS ≤ 20th percentile and/or pLI score ≥ 0.9) and (d) likely deleterious variants in genes with a predicted interaction with NRXN1 in STRING. We identified one de novo stop‐gain in *CDC25C* (NP_073720.1:p.(Ser143Xaa)) and de novo missense in *WASHC5* (NP_001317538.1:p.(Asp254Gly)), 2 homozygous missense variants in *ZFP37* and *DCLRE1A* and two compound heterozygous variants in *IFT80*, and 5 missense variants in SFARI ASD candidate genes intolerant to mutation (*CNTNAP5, ERBIN, SYNE, HERC2 and TANC2*); all of them, except for *TANC2*, were of paternal origin in brain expressed genes. Furthermore, we highlighted 4 genes with damaging missense variants that are predicted to interact with *NRXN1* (*CNTNAP5, TULP1, NCAN* and *KIRREL2*) (Table 2). Among them, the missense variant in *CNTNAP5* is likely to exert a significant role, given that *CNTNAP5* is itself a previously known ASD candidate gene, likely to be intolerant to mutations (RVIS percentile = 8.4) (Figure 2). Finally, we investigated the mother's burden of rare variants in these risk categories and, although we are not able to identify de novo and compound heterozygous variants, there is still a higher number of variants in the proband compared with the mother (11 vs 6 variants) (Table S2).

**Table 2:** Rare LGD and predicted damaging missense variants in four‐risk categories identified in the ASD proband

Gene base change	Identified Variants						SFARI gene score	pLI score	RVIS percentile	PSD/Synaptic/ Brain expressed^20^, ^21^, ^22^
	AminoAcid Change	Effect	Inheritance	dbSNP	MAF in gnomAD (exome)	Gene				
a) De novo variants										
**NC_000005.9:g.137627774G>T**	**NP_073720.1:p.(Ser143Xaa)**	**Stopgain**	**De Novo**			***CDC25C***		3.01E‐08	46.96	**Brain expressed**
**NC_000008.10:g.126079907T>C**	**NP_001317538.1:p.(Asp254Gly)**	**Missense**	**De Novo**			***WASHC5***		4.81E‐13	1.94	**PSD/ Brain expressed**
b) Homozygous and compound heterozygous variants										
NC_000003.11:g.160000321A>T	NP_001177171.1:p.(Asp350Glu)	Missense	Maternal			*IFT80*		1.44E‐10	50.94	Brain expressed
NC_000003.11:g.160037568T>C	NP_001177171.1:p.(Thr176Ala)	Missense	Paternal	rs146065418	0.0004781	*IFT80*		1.44E‐10	50.94	Brain expressed
NC_000009.11:g.115812140G>T	NP_001269444.1:p.(Leu64Met)	Missense	Paternal/ Maternal	rs151180938	0.0029	*ZFP37*		4.11E‐09	45.02	Brain expressed
NC_000010.10:g.115602192T>A	NP_055696.3:p.(Ile859Phe)	Missense	Paternal/ Maternal	rs11196530	0.002732	*DCLRE1A*		4.68E‐08	70.92	Brain expressed
c) Variants in SFARI Gene intolerant to mutation^a^										
NC_000002.11:g.125660581G>A	NP_570129.1:p.(Ala1186Thr)	Missense	Paternal	rs114400050	0.00231	*CNTNAP5*	3	0.10	8.42	Brain expressed
NC_000005.9:g.65321695C>G	NP_001006600.1:p.(Leu304Val)	Missense	Paternal	rs148121803	0.0001956	*ERBIN*	2	0.99	63.5	PSD/ Synaptic/ Brain expressed
NC_000006.11:g.152642393C>G	NP_149062.1:p.(Asp5335His)	Missense	Paternal			*SYNE1*	3‐S	3.75E‐27	7.64	PSD/ Brain expressed
NC_000015.9:g.28479425G>A	NP_004658.3:p.(Leu1337Phe)	Missense	Paternal	rs145594989	0.002391	*HERC2*	S	1	0.16	Brain expressed
NC_000017.10:g.61498241A>G	NP_079461.2:p.(Tyr1633Cys)	Missense	Maternal	rs765761955	0.000004011	*TANC2*	1	1	0.33	Brain expressed^b^
d) Variants in genes interacting with NRXN1^#^										
NC_000002.11:g.125660581G>A	NP_570129.1:p.(Ala1186Thr)	Missense	Paternal	rs114400050	0.00231	*CNTNAP5*	3	0.10	8.42	Brain expressed
NC_000006.11:g.35467787T>C	NP_001276324.1:p.(Lys436Arg)	Missense	Maternal	rs62636511	0.00001989	*TULP1*		0.81	65.05	
NC_000019.9:g.19335134C>T	NP_004377.2:p.(Arg224Cys)	Missense	Maternal			*NCAN*		0.15	6.15	PSD/ Synaptic/ Brain expressed
NC_000019.9:g.36351545C>A	NP_001316459.1:p.(Arg252Ser)	Missense	Paternal	rs73928337	0.001664	*KIRREL2*		8.95E‐06	93.31	Brain expressed

^a^ pLI score ≥ 0.9 and/or RVIS percentile ≤ 20 (underlined); #Predicted protein‐protein interaction in STRING.

^b^ Brain expressed according to^51^; Variants validated by Sanger sequencing are highlighted in bold.

### 3.4 Mitochondrial DNA analyses

We carried out deep sequencing of the entire mtDNA in the ASD proband and her parents (Table S3 and S4). Both the proband and her mother showed all defining variants of haplogroup H13a1a1 of European ancestry, whereas the father's variants identified the haplogroup L2c2b1b background of African ancestry. None of the rare variants were previously reported (private or unique to an individual) or predicted as pathogenic.

Taking advantage of the high mean coverage in sequencing (16721X in the proband, 12090X in the mother and 19027X in the father), we were able to detect variants with a low‐level heteroplasmy (between 0.2% and 15%), in all three individuals (Table S3 and S4). Two of these were present in the proband and inherited from the mother: the synonymous variant NC_012920.1(MT‐ND2):m.4847C>T and the non‐coding NC_012920.1(MT‐HV1):m.16092T>C , one of the hypervariable domains of the control region. The heteroplasmy of these variants was higher in the proband (13.6% and 12.7%, respectively) as compared with the mother (2.2% and 2.1%, respectively). Furthermore, other five variants with a very low‐level of heteroplasmy (0.2%‐0.7%) were found exclusively in the proband: the NC_012920.1(MT‐RNR2):m.1906G>A, NC_012920.1(MT‐RNR2):m.2009G>A and NC_012920.1(MT‐TL1):m.3242G>A, which were never reported in over 50.000 sequence (GenBank), whereas the missense NC_012920.1(MT‐CO3):p.(Val208Ile) and the non‐coding NC_012920.1(MT‐HV1):m.16348C>T variants were previously reported three and six times, respectively. The missense NC_012920.1(MT‐CO3):p.(Val208Ile) variant, even if never reported as pathogenic for any disease, was predicted to be deleterious. The mother presented only one private low‐level heteroplasmic variant, the missense NC_012920.1(MT‐ND5):p.(Phe326Ser,) (2.2%), never reported in GenBank and also predicted to be deleterious. The father presented four low‐level heteroplasmic variants: the missense NC_012920.1(MT‐CYB):p.(Gly251Asp) (0.6%), reported 6 time in GenBank and predicted to be deleterious, and the non‐coding variants NC_012920.1(MT‐HV1):m.16192C>T (0.4%), NC_012920.1(MT‐HV1):m.16256C>T (0.8%), NC_012920.1(MT‐HV1):m.16291C>T (0.9%), very frequent in GenBank (2029, 1645, 1341, respectively).

To verify the remote possibility of biparental inheritance, as recently reported,^34^ we also compared the proband and her father mtDNA sequences. With the exception of the reference sequence private variants,^35^ the proband and her father did no share any other variants, not even at low‐level heteroplasmy (Table S3 and S4).

Last, the mtDNA content of the proband and her parents was comparable to the range of age‐matched healthy individuals (Figure S2).

## 4 DISCUSSION

In this study, we identified an intragenic *NRXN1* deletion in a female proband with ASD, who has inherited it from the unaffected mother. We have thus characterized rare variants in the nuclear and mitochondrial genome of this family, in order to investigate their contribution towards the manifestation of the ASD phenotype in *NRXN1* deletion carriers.

The *NRXN1* deletion identified in this family can be classified as a 3’deletion,^8^ as it overlaps exons from 7 to 23 (NM_001135659.2). The deletion gives rise to a putative in‐frame transcript, lacking the majority of NRXN1 protein domains (from Gly311 to Leu1445), specifically all α‐neurexin LNS‐domains (laminin/neurexin/sex hormone‐binding globulin domains) except the first one, and the two intercalated epidermal growth factor (EGF)‐like domains, while maintaining the transmembrane and intracellular C‐terminal domain (Figure 1B). Moreover, the deletion impacts the canonical splice sites (SS2 to SS6), including SS4, thought to represent a key mechanism for the regulation of NRXN‐ligand interactions at synapses.^9^ As the 3’deletion identified in our proband is in‐frame, it is possible that the phenotypic effect of this deletion may arise by two concurrent mechanisms: haploinsufficiency due to lack of wild‐type *NRXN1*α isoforms, and a dominant‐negative activity of the mutant splice isoform, as suggested by a recent study using induced pluripotent stem cell (hiPSC)‐derived neurons from subjects with heterozygous intragenic deletions.^36^

The proband's phenotype is mainly compatible with clinical features of 3’ deletion carriers, as the girl with ASD has macrocephaly (10.3% of 3’deletion carriers had macrocephaly in comparison with only 1.2% of 5’ deletion carriers) and DD (more frequently presents in probands with 3’ deletions).^8^ Given the importance of collecting phenotype information also on parents to look for possible endophenotypes, we have also evaluated both parents for the presence of broad autism phenotype signs, but scores in questionnaires (SCDC, BAPQ) were not consistent with any ASD traits in either of them.

Whole‐exome sequencing analysis in all family members identified a higher number of rare coding variants in the ASD child in comparison with the *NRXN1* deletion‐transmitting mother and this difference remains statistically significant if we restrict the analysis to rare likely deleterious mutations only, suggesting that additional rare variants may contribute collectively to push the genetic liability beyond the threshold for ASD. Moreover, a significant interaction enrichment was detected among genes with damaging variants (CNVs and SNVs) in the proband, supporting a cumulative effect of interacting genes affected by mutations to the phenotype. Interestingly, among the *NRXN1* interactions detected by STRING in the ASD proband, there is a predicted interaction with *CNTNAP5*, a functionally intolerant gene previously identified as an ASD candidate gene, which harbours a paternally inherited putative damaging missense variant. *CNTNAP5* encodes for contactin‐associated protein‐like 5, a member of the neurexin family involved in cell adhesion and intercellular communication in the vertebrate nervous system.^37^ *CNTNAP5* is classified as a suggestive candidate gene for ASD (SFARI score 3), as a rare deletion and missense variants in *CNTNAP5* have been identified in subjects with ASD.^38^ Similarly to this report, a previous study identified a maternally inherited missense variant in *CNTNAP5* segregating with a *NRXN3* paternal deletion in two ASD siblings,^39^ supporting a combined role of neurexins and contactin‐associated proteins in ASD risk.

In addition to *CNTNAP5*, damaging missense variants were identified in four other mutation intolerant genes, previously implicated in ASD: *TANC2*, *ERBIN*, *SYNE1* and *HERC2*.^40^, ^41^, ^42^, ^43^, ^44^, ^45^, ^46^, ^47^, ^48^, ^49^, ^50^ While *TANC2* and *ERBIN* have been involved in idiopathic ASD susceptibility with high confidence (SFARI score 1 and 2, respectively), *SYNE1* and *HERC2* gene have been mostly involved in syndromic ASD. Moreover, *TANC2* and *ERBIN* are interesting functional candidate genes. *TANC2* is highly expressed in the human developing brain and encodes for a postsynaptic scaffold protein involved in dendritic spines and excitatory synapses regulation.^51^ *ERBIN* encodes a postsynaptic protein which binds ERBB2 playing an important role during brain development and regulation of synaptic plasticity in the adult brain.^52^ *ERBIN* is also implicated in dendritic morphogenesis by regulating localization and function of δ‐Catenin in hippocampal neurons.^53^

Two de novo novel putative damaging variants were also identified in the proband: a stop‐gain variant located on *CDC25C* exon 5 (NP_073720.1:p.(Ser143Xaa)) and a predicted damaging missense variant in *WASHC5* exon 9 (NP_001317538.1:p.(Asp254Gly)). Although neither of them have been previously implicated in ASD, both of them are expressed in the brain^22^ and therefore they could contribute to the proband phenotype. It should be noted that heterozygous missense variants in *WASHC5* are associated with autosomal dominant spastic paraplegia 8 (SPG8), a progressive upper‐motor neurodegenerative disease,^54^ while biallelic pathogenic variants are also associated with Ritscher‐Schinzel Syndrome, a clinically recognizable condition characterized by distinctive craniofacial features, cerebellar defects and cardiovascular malformations.^55^ The *WASHC5* variant (NP_001317538.1:p.(Asp254Gly)) is predicted to be deleterious, is novel and it is located in the spectrin‐like repeat domain, thus, we cannot rule out a pathological role for this mutation for a spastic paraplegia phenotype, which usually has on onset in adult life. Moreover, although the proband did not show typical dysmorphic craniofacial features of individuals with Ritscher‐Schinzel, she presented macrocrania.

Recessive‐acting variants were identified in three genes (*ZFP37*, *DCLRE1A* and *IFT80*), all of which are brain‐expressed^22^ but neither of them have been previously implicated in neurodevelopmental phenotypes.

Finally, the proband did not carry any pathogenic mutation in her mtDNA. However, we found five variants with low‐level heteroplasmy (ranging from 0.2% to 0.7%) that were absent in the mother, and two maternally inherited variants, which increased their heteroplasmic load in the proband as compared to the mother. The burden of low‐level heteroplasmic mtDNA variants, both inherited or de novo, also known as universal heteroplasmy,^56^ might contribute to the risk of developing ASD, but further analyses on large cohorts are needed to validate this hypothesis. The mtDNA copy number was also uninformative.

**Figure 3:**
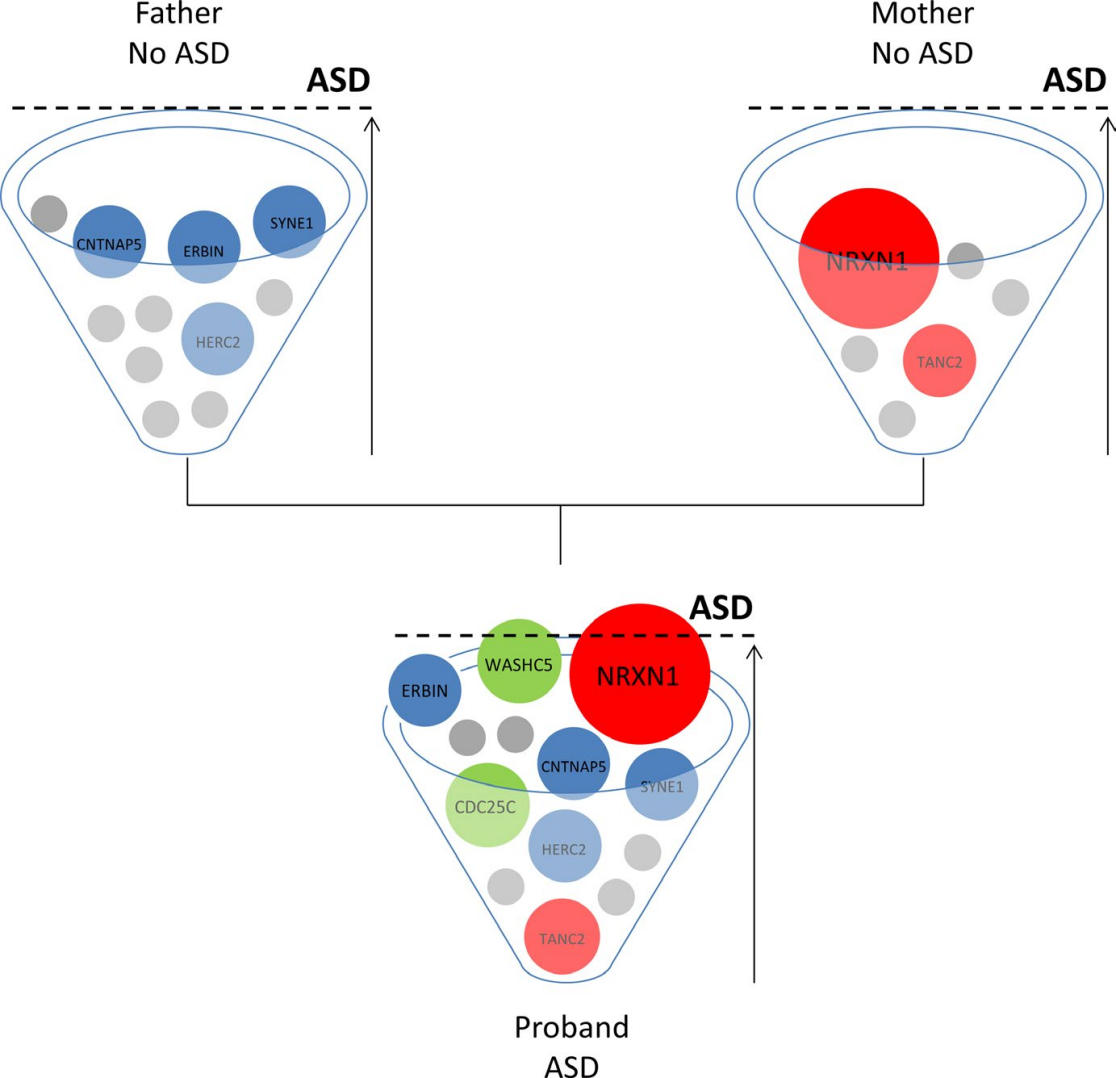
Multi‐factorial threshold model for ASD in this family. Each family member has an ASD risk cup with balls representing risk variants that contribute to ASD with variable degrees of impact. In both parents, the burden of risk variants is not enough to develop ASD, while in the child the ASD threshold is reached as a combination of strong and weak, inherited and de novo genetic variants. The *NRXN1* deletion is depicted as a strong, primary contributing factor to reaching the ASD threshold in the ASD child, but not sufficient alone to develop ASD in the deletion carrier mother^57^

In conclusion, we have characterized a trio family in which a large 3’ exonic *NRXN1* deletion is transmitted from an unaffected mother to a child with ASD. Exonic *NRXN1* deletions represent the prototype of incomplete penetrant ASD‐associated susceptibility variants as they are often inherited from unaffected or mildly affected parents, but they are still considered pathogenic. The key finding is the presence of an increased burden of exonic rare variants in the affected proband compared to the unaffected deletion‐transmitting mother, supporting the hypothesis that the *NRXN1* deletion sensitizes the genome to a clinical manifestation, but other genetic contributors are necessary to cross the threshold for a phenotypic manifestation (Figure 3).^57^ Moreover, the reduced penetrance of the *NRXN1* deletion in the unaffected mother is consistent with a female protective effect: females would require an excess burden of deleterious CNVs and SNVs to reach the ASD diagnostic threshold.^58^ Therefore, in this family, the paternal‐inherited rare variants in ASD‐related or functionally constrained genes and de novo rare variants identified in the proband may have additive effects acting on a sensitized background caused by haploinsufficiency and/or a dominant‐negative activity at the *NRXN1* locus. This observation is in line with the hypothesis that the determinants of psychiatric traits are multifactorial even in the context of a large‐effect variant. These modifying determinants may include the genetic background of common polygenic variants as well as rare variants. It has been recently reported that the increased burden of rare likely deleterious variants enhances the expression of neurodevelopmental phenotypes in probands with 16p21.1 deletions and in probands with other gene disruptive variants compared with their carrier family members^59^; hence, in this study, we have focused our attention on the background of rare variants. Our results are consistent with the hypothesis that the burden of rare variants contributes in defining the phenotypic trajectory in carriers of a large‐effect variant, thus, this may represent a widespread mechanism that modulates the penetrance and the expressivity of disease‐associated variants. However, it has been shown that also common polygenic variation contributes additively to ASD risk, even in cases that carry a strongly acting variant.^60^, ^61^ A limitation to our study is therefore that we were not able to test the potential contribution of common variation in modulating the penetrance of the *NRXN1* deletion in this family.

Further investigation in a large dataset will be necessary to properly evaluate the cumulative effects of rare deleterious and common variants to ASD risk in *NRXN1* deletion carriers; family‐based samples will be particularly informative, as they allow intrafamilial comparison of phenotypic features and inheritance pattern of specific variants.

This study underlines the importance of a comprehensive assessment of the genomic landscape of ASD individuals even when a ‘likely pathogenic’ variant has been already identified, as it is apparent that multiple rare variants contribute in conjunction to the overall genetic risk and the final clinical outcome. It is time to move from a genetic to a genomic perspective, shifting from a single variant analysis to an integrated view of many variants of different origin (nuclear and mitochondrial), types (CNVs and SNVs), inheritance pattern (de novo and inherited), frequency (rare and common, heteroplasmic and homoplasmic) and effect sizes, considering the role of protein‐protein interactions.

## CONFLICT OF INTEREST

The authors confirm that there are no conflicts of interest.

## AUTHOR CONTRIBUTIONS

**Cinzia Cameli:** Data curation (equal); Formal analysis (equal); Investigation (equal); Software (equal); Validation (lead); Visualization (equal); Writing‐original draft (supporting). **Marta Viaggiano:** Data curation (equal); Formal analysis (equal); Investigation (equal); Software (equal); Validation (supporting); Visualization (equal). **Magali Jane Rochat:** Formal analysis (equal); Funding acquisition (equal); Investigation (equal); Resources (supporting); Visualization (equal); Writing‐original draft (supporting). **Alessandra Maresca:** Formal analysis (equal); Funding acquisition (equal); Investigation (equal); Writing‐original draft (supporting). **Leonardo Caporali:** Formal analysis (equal); Investigation (equal); Writing‐original draft (supporting). **Claudio Fiorini:** Formal analysis (equal); Investigation (equal); Writing‐original draft (supporting). **Flavia Palombo:** Investigation (equal); Software (equal). **Pamela Magini:** Investigation (equal). **Renée Concetta Duardo:** Investigation (equal). **Fabiola Ceroni:** Validation (supporting). **Maria Cristina Scaduto:** Investigation (equal); Resources (supporting). **Annio Posar:** Investigation (equal); Resources (supporting). **Marco Seri:** Supervision (equal). **Valerio Carelli:** Methodology (equal); Project administration (equal); Supervision (equal); Writing‐review & editing (equal). **Paola Visconti:** Methodology (equal); Project administration (equal); Resources (lead); Supervision (equal); Writing‐review & editing (equal). **ELENA BACCHELLI:** Conceptualization (equal); Formal analysis (equal); Funding acquisition (equal); Methodology (equal); Project administration (equal); Software (equal); Supervision (equal); Visualization (equal); Writing‐original draft (lead); Writing‐review & editing (equal). **Elena Maestrini:** Conceptualization (equal); Methodology (equal); Project administration (equal); Supervision (equal); Writing‐review & editing (equal).

## Supporting information

Supplementary Material

## Data Availability

The data that support the findings of this study are available in the supplementary material of this article.
