# Supplementary material for: An increased burden of rare exonic variants in NRXN1 microdeletion carriers is likely to enhance the penetrance for autism spectrum disorder
Source: J Cell Mol Med. 2021 Jan 21;25(5):2459–70. doi: 10.1111/jcmm.16161 (PMC7933976; doi:10.1111/jcmm.16161)
Supplement: Supplementary file 1 — Supplementary Material [file JCMM-25-2459-s001.docx]

**An increased burden of rare exonic variants in *NRXN1* microdeletion carriers is likely to enhance the penetrance for autism spectrum disorder.**

**(Supplementary Material)**

Cinzia Cameli^1¶^, Marta Viggiano^1¶^, Magali J. Rochat^2^, Alessandra Maresca^3^, Leonardo Caporali^3^, Claudio Fiorini^3,4^, Flavia Palombo^3^, Pamela Magini^5^, Renée C. Duardo^1^, Fabiola Ceroni^6^, Maria C. Scaduto^2^, Annio Posar^2,4^, Marco Seri^5^, Valerio Carelli^3,4^, Paola Visconti^2^, Elena Bacchelli^1^*, Elena Maestrini^1^*

^1^ Department of Pharmacy and Biotechnology, University of Bologna, Bologna, Italy

^2^ IRCCS Istituto delle Scienze Neurologiche di Bologna, UOC Neuropsichiatria Infantile, Bologna, Italia.

^3^ IRCCS Istituto delle Scienze Neurologiche di Bologna, UOC Clinica Neurologica, Bologna, Italia.

^4^ Unit of Neurology, Department of Biomedical and Neuromotor Sciences (DIBINEM), University of Bologna, Bologna, Italy.

^5^ Unit of Medical Genetics, Department of Medical and Surgical Sciences, Policlinico St. Orsola-Malpighi Hospital, University of Bologna, Bologna, Italy.

^6^ Faculty of Health and Life Sciences, Oxford Brookes University, Oxford OX3 0BP, UK;

* Corresponding authors

E-mail: elena.bacchelli@unibo.it (EB)

Tel: +39-051-2094085 (EB)

E-mail: elena.maestrini@unibo.it (EM)

Tel: +39-051-2094178 (EM)

^¶^These authors contributed equally to this work.


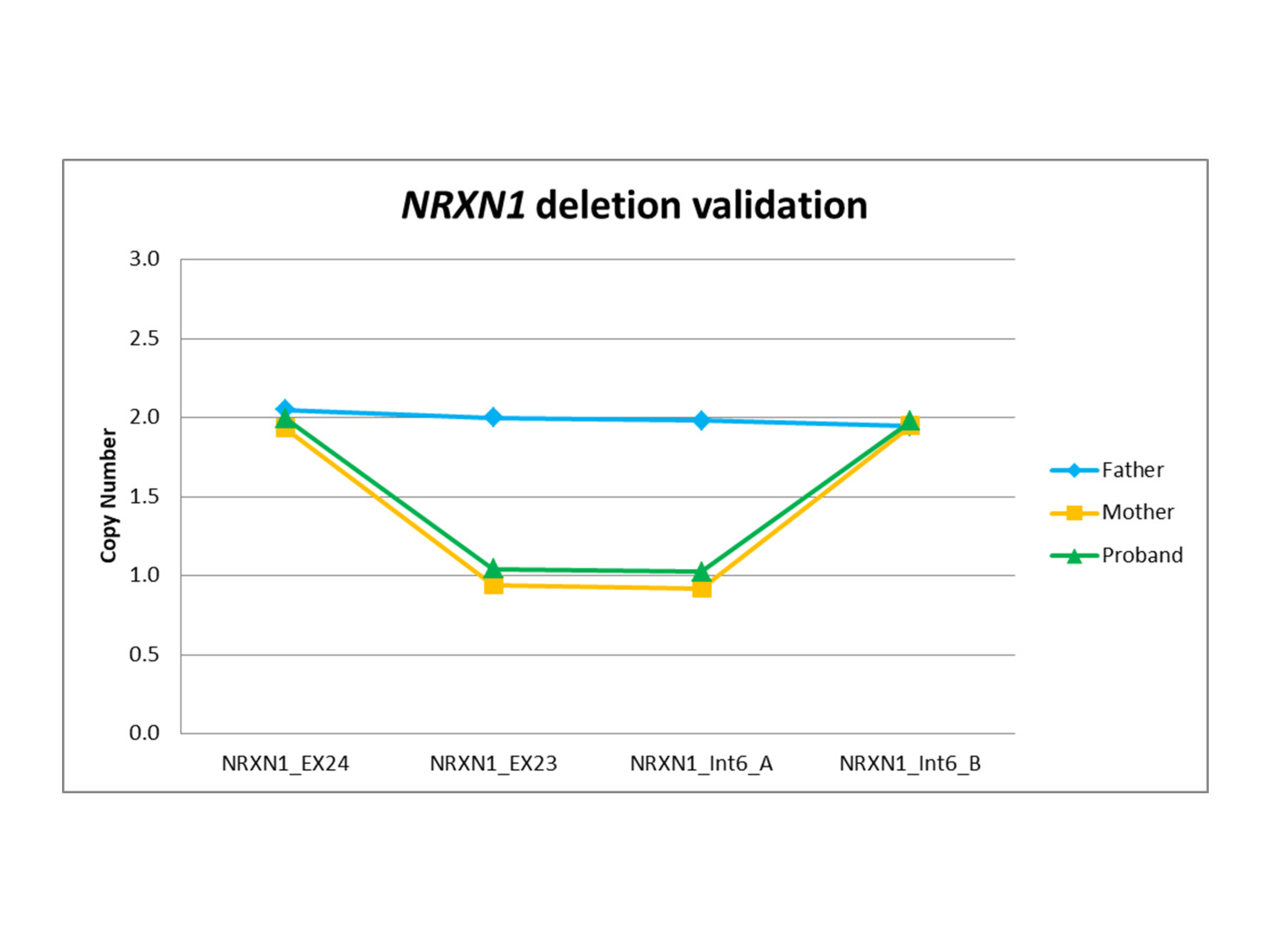


**Supplementary Figure 1:** Validation and segregation analysis of *NRXN1* deletion.





**Supplementary Figure 2:** mtDNA content assessed in proband and her parents. Two different age-matched control groups were used to compare parents and probands. Anova test showed no significant differences between control groups and the three individuals of this family. CTRLS adults n=86, CTRLS children n=27.

**Supplementary table S1.** Rare CNVs identified in the ASD proband by SurePrint G3 Unrestricted CGH ISCA v2, 8x60K (Agilent Technologies)

| **Chromosome band** | **Start** | **End** | **Estimated size (bp)** | **CNV type** | **Gene** | **Class^§^** |
| --- | --- | --- | --- | --- | --- | --- |
| 1q42.13 | 230,190,853 | 230,340,358 | 149,505 | Duplication | *GALNT2* | Uncertain significance |
| **2p16.3** | **50,170,766** | **50,982,172** | **811,406** | **Deletion** | ***NRXN1*** | **Pathogenic** |
| 6p25.1 | 4,269,700 | 4,437,880 | 168,180 | Deletion | *-* | Likely benign |
| 9q22.1 | 91,353,932 | 91,492,516 | 138,584 | Deletion | *MIR4289* | Uncertain significance: likely benign |

§Classification based on Kearney et al.^1^

| **Identified Variants** | | | | | | **SFARI gene score** | **pLI score** | **RVIS percentile** | **PSD/Synaptic/**  **Brain expressed^2-4^** |
| --- | --- | --- | --- | --- | --- | --- | --- | --- | --- |
| **Gene Base Change** | **AminoAcid Change** | **Effect** | **dbSNP** | **MAF in gnomAD (exome)** | **Gene** |  |  |  |  |
| ***Variants in SFARI Gene intolerant to mutation**** | | | | | | | | | |
| NC_000017.10:g.61498241A>G | NP_079461.2:p.(Tyr1633Cys) | Missense | rs765761955 | 0.000004011 | *TANC2* | 1 | 1 | 0.33 | Brain expressed^§^ |
| ***Variants in genes interacting with NRXN1^#^*** | | | | | | | | | |
| NC_000006.11 :g.131277575C>G | NP_001129026.1:p.(Gln17His) | Missense |  |  | *EPB41L2* |  | 0.99 | 76 | PSD/ Synaptic/ Brain expressed |
| NC_000006.11 :g.35467787T>C | NP_001276324.1:p.(Lys436Arg) | Missense | rs62636511 | 0.00001989 | *TULP1* |  | 0.81 | 65.05 |  |
| NC_000011.9 :g.85468708G>A | NP_001156423.1:p.(Arg21Trp) | Missense | rs149582759 | 0.003446 | *SYTL2* |  | 2.12E-19 | 91.61 | Synaptic/ Brain expressed |
| NC_000017.10 :g.73509815G>A | NP_065804.2:p.(Ala24Val) | Missense | rs745781743 | 0.00001994 | *CASKIN2* |  | 1.00 | 72.1 | Synaptic/ Brain expressed |
| NC_000019.9 :g.19335134C>T | NP_004377.2:p.(Arg224Cys) | Missense |  |  | *NCAN* |  | 0.15 | 6.15 | PSD/ Synaptic/ Brain expressed |

**Table S2:** Rare LGD and predicted damaging missense variants in selected risk categories identified in the *NRXN1* deletion-transmitting unaffected mother

*pLI score ≥0.9 and/or RVIS percentile ≤ 20 (underlined); #Predicted protein-protein interaction in STRING; § Brain expressed according to Han et al^5^.

**Supplementary table 3.** Mitochondrial variants in proband and her mother.

| **Position** | **Ref** | **Alt** | **Mother** | **Proband** | **Gene** | **Effect** | **Pathogenicity** |  |
| --- | --- | --- | --- | --- | --- | --- | --- | --- |
| **Variants diagnostic for haplogroup H13a1a1** | | | | | | | | |
| 263^a^ | A | G | 100% | 100% | MT-HV2 | - | - |  |
| 750^a^ | A | G | 100% | 100% | MT-RNR1 | - | - |  |
| 1438^a^ | A | G | 100% | 100% | MT-RNR1 | - | - |  |
| 2259 | C | T | 100% | 100% | MT-RNR2 | - | - |  |
| 4745 | A | G | 100% | 100% | MT-ND2 | syn | - |  |
| 4769^a^ | A | G | 100% | 100% | MT-ND2 | - | - |  |
| 7337 | G | A | 100% | 100% | MT-CO1 | - | - |  |
| 8860^a^ | A | G | 100% | 100% | MT-ATP6 | Thr112Ala | 0.14 |  |
| 13326 | T | C | 100% | 100% | MT-ND5 | syn | - |  |
| 13680 | C | T | 100% | 100% | MT-ND5 | syn | - |  |
| 14872 | C | T | 100% | 100% | MT-CYB | syn | - |  |
| 15326^a^ | A | G | 100% | 100% | MT-CYB | Thr194Ala | 0.12 |  |
| **Private variants** | | | | | | | | |
| 204 | T | C | 100% | 100% | MT-HV2 | - | - |  |
| 7987 | A | G | 100% | 100% | MT-CO2 | syn | - |  |
| 12397 | A | G | 100% | 100% | MT-ND5 | Thr21Ala | 0.26 |  |
| 16266 | C | T | 100% | 100% | MT-HV1 | - | - |  |
| **Inherited low-level variants** | | | | | | | | **GB** |
| 4847 | C | T | 2,2% | 13,6% | MT-ND2 | syn | - | 4 |
| 16092 | T | C | 2,1% | 12,8% | MT-HV1 | - | - | 685 |
| **Private low-level variants in proband** | | | | | | | | |
| 1906 | G | A |  | 0,4% | MT-RNR2 | - | - | 0 |
| 2009 | G | A |  | 0,6% | MT-RNR2 | - | - | 0 |
| 3242 | G | A |  | 0,3% | MT-TL1 | - | - | 0 |
| 9828 | G | A |  | 0,2% | MT-CO3 | Val208Ile | 0.68 | 3 |
| 16348 | C | T |  | 0,7% | MT-HV1 | - | - | 6 |
| **Private low-level variants in mother** | | | | | | | | |
| 13313 | T | C | 2,2% |  | MT-ND5 | Phe326Ser | 0.74 | 0 |

GB= number of sequences found on GenBank (n=50.175 full length mtDNA sequences)

aPrivate variants of reference sequence rCRS NC_012920.1 (Andrews et al, 1999).

**Supplementary table S4.** Mitochondrial variants in probands’ father.

| **Position** | **Ref** | **Alt** | **Father** | **Gene** | **Effect** | **Pathogenicity** |  |
| --- | --- | --- | --- | --- | --- | --- | --- |
| **Variants diagnostic for haplogroup L2c2b1b** | | | | | | | |
| 73 | A | G | 100% | MT-HV2 | - | - |  |
| 93 | A | G | 100% | MT-HV2 | - | - |  |
| 146 | T | C | 100% | MT-HV2 | - | - |  |
| 150 | C | T | 100% | MT-HV2 | - | - |  |
| 152 | T | C | 100% | MT-HV2 | - | - |  |
| 182 | C | T | 100% | MT-HV2 | - | - |  |
| 183 | A | G | 100% | MT-HV2 | - | - |  |
| 195 | T | C | 100% | MT-HV2 | - | - |  |
| 198 | C | T | 100% | MT-HV2 | - | - |  |
| 263^a^ | A | G | 100% | MT-HV2 | - | - |  |
| 325 | C | T | 100% | MT-HV2 | - | - |  |
| 680 | T | C | 100% | MT-RNR1 | - | - |  |
| 709 | G | A | 100% | MT-RNR1 | - | - |  |
| 750^a^ | A | G | 100% | MT-RNR1 | - | - |  |
| 769 | G | A | 100% | MT-RNR1 | - | - |  |
| 1018 | G | A | 100% | MT-RNR1 | - | - |  |
| 1040 | T | C | 100% | MT-RNR1 | - | - |  |
| 1438^a^ | A | G | 100% | MT-RNR1 | - | - |  |
| 1442 | G | A | 100% | MT-RNR1 | - | - |  |
| 2332 | C | T | 100% | MT-RNR2 | - | - |  |
| 2416 | T | C | 100% | MT-RNR2 | - | - |  |
| 2706 | A | G | 100% | MT-RNR2 | - | - |  |
| 3200 | T | A | 100% | MT-RNR2 | - | - |  |
| 3594 | C | T | 100% | MT-ND1 | syn | - |  |
| 4104 | A | G | 100% | MT-ND1 | - | - |  |
| 4769^a^ | A | G | 100% | MT-ND2 | - | - |  |
| 7028 | C | T | 100% | MT-CO1 | - | - |  |
| 7256 | C | T | 100% | MT-CO1 | syn | - |  |
| 7521 | G | A | 100% | MT-TD | - | - |  |
| 7624 | T | A | 100% | MT-CO2 | syn | - |  |
| 8206 | G | A | 100% | MT-CO2 | syn | - |  |
| 8567 | T | C | 100% | MT-ATP8 | Ser68Pro | 0.82 |  |
| 8701 | A | G | 100% | MT-ATP6 | Thr59Ala | 0.07 |  |
| 8772 | T | C | 100% | MT-ATP6 | - | - |  |
| 8860^a^ | A | G | 100% | MT-ATP6 | Thr112Ala | 0.14 |  |
| 9063 | A | G | 100% | MT-ATP6 | - | - |  |
| 9221 | A | G | 100% | MT-CO3 | syn | - |  |
| 9540 | T | C | 100% | MT-CO3 | syn | - |  |
| 10115 | T | C | 100% | MT-ND3 | syn | - |  |
| 10398 | A | G | 100% | MT-ND3 | Thr114Ala | 0.04 |  |
| 10790 | T | C | 100% | MT-ND4 | syn | - |  |
| 10873 | T | C | 100% | MT-ND4 | - | - |  |
| 11719 | G | A | 100% | MT-ND4 | - | - |  |
| 11944 | T | C | 100% | MT-ND4 | - | - |  |
| 12236 | G | A | 100% | MT-TS2 | - | - |  |
| 12705 | C | T | 100% | MT-ND5 | syn | - |  |
| 13590 | G | A | 100% | MT-ND5 | syn | - |  |
| 13650 | C | T | 100% | MT-ND5 | - | - |  |
| 13928 | G | C | 100% | MT-ND5 | Ser531Thr | 0.32 |  |
| 13958 | G | C | 100% | MT-ND5 | Gly541Ala | 0.38 |  |
| 14766 | C | T | 100% | MT-CYB | Thr7Ile | 0.09 |  |
| 15110 | G | A | 100% | MT-CYB | Ala122Thr | 0.11 |  |
| 15217 | G | A | 100% | MT-CYB | - | - |  |
| 15301 | G | A | 100% | MT-CYB | syn | - |  |
| 15313 | T | C | 100% | MT-CYB | syn | - |  |
| 15326^a^ | A | G | 100% | MT-CYB | Thr194Ala | 0.12 |  |
| 15849 | C | T | 100% | MT-CYB | Thr368Ile | 0.09 |  |
| 16223 | C | T | 100% | MT-HV1 | - | - |  |
| 16264 | C | T | 100% | MT-HV1 | - | - |  |
| 16278 | C | T | 100% | MT-HV1 | - | - |  |
| 16311 | T | C | 100% | MT-HV1 | - | - |  |
| 16390 | G | A | 100% | MT-CR | - | - |  |
| 16519 | T | C | 100% | MT-CR | - | - |  |
| **Private variants** | | | | | | | |
| 7080 | T | C | 100% | MT-CO1 | Phe393Leu | 0.7 |  |
| 16069 | C | T | 100% | MT-HV1 | - | - |  |
| **Private low-level variants** | | | | | | | **GB** |
| 15498 | G | A | 0,6% | MT-CYB | Gly251Asp | 0.69 | 13 |
| 16192 | C | T | 0,4% | MT-HV1 | - | - | 2029 |
| 16256 | C | T | 0,8% | MT-HV1 | - | - | 1645 |
| 16291 | C | T | 0,9% | MT-HV1 | - | - | 1341 |

GB= number of sequences found on GenBank (n=50.175 full length mtDNA sequences)

aPrivate variants of reference sequence rCRS NC_012920.1^6^

**References**

1. Kearney HM, Thorland EC, Brown KK, Quintero-Rivera F, South ST. American College of Medical Genetics standards and guidelines for interpretation and reporting of postnatal constitutional copy number variants. *Genet Med.* 2011;13(7):680-685.

2. Bayes A, van de Lagemaat LN, Collins MO, et al. Characterization of the proteome, diseases and evolution of the human postsynaptic density. *Nat Neurosci.* 2011;14(1):19-21.

3. Jansen A, Dieleman GC, Smit AB, et al. Gene-set analysis shows association between FMRP targets and autism spectrum disorder. *Eur J Hum Genet.* 2017;25(7):863-868.

4. Kang HJ, Kawasawa YI, Cheng F, et al. Spatio-temporal transcriptome of the human brain. *Nature.* 2011;478(7370):483-489.

5. Han S, Nam J, Li Y, et al. Regulation of dendritic spines, spatial memory, and embryonic development by the TANC family of PSD-95-interacting proteins. *J Neurosci.* 2010;30(45):15102-15112.

6. Andrews RM, Kubacka I, Chinnery PF, Lightowlers RN, Turnbull DM, Howell N. Reanalysis and revision of the Cambridge reference sequence for human mitochondrial DNA. *Nat Genet.* 1999;23(2):147.
